# Effect of organic ligand-decorated ZnO nanoparticles as a cathode buffer layer on electricity conversion efficiency of an inverted solar cell

**DOI:** 10.1039/c7ra11902j

**Published:** 2018-01-04

**Authors:** Alireza Samavati, Zahra Samavati, A. F. Ismail, M. H. D. Othman, Mukhlis A. Rahman, I. S. Amiri

**Affiliations:** Advanced Membrane Technology Research Centre (AMTEC), Universiti Teknologi Malaysia 81310 Skudai Johor Malaysia afauzi@utm.my; Computational Optics Research Group, Ton Duc Thang University Ho Chi Minh City Vietnam irajsadeghamiri@tdt.edu.vn; Faculty of Applied Sciences, Ton Duc Thang University Ho Chi Minh City Vietnam

## Abstract

Efficiency improvement of the industrial scale solar cells to capture sunlight as an important renewable energy source is attracting significant attention to prevent the consumption of a finite supply of unsustainable fossil fuels. ZnO nanoparticles decorated with an imine-linked receptor have been used in the fabrication of a photocathode based on dye-sensitized solar cells for the purpose of photovoltaic efficiency enhancement. Various characterization techniques have been employed to investigate the structural, morphological, and optical behaviors of the solar cell having ZnO nanoparticles and ZnO nanoparticles decorated with an organic ligand as a photocathode layer. The decorated nanoparticles have a stable wurtzite structure and an average grain size of ∼45 nm, confirmed by the TEM image and XRD through the Scherrer equation. The ZnO sample emits wide peaks in the visible range, and the emission intensity of the ZnO-DOL sample increases along with a red-shift (0.38 eV) in the band gap. This shift can be explained using deep level transition, surface plasmon energy of a surfactant, and coupling of ZnO with local surface plasmon energy. UV-vis absorption spectra together with photoluminescence spectra confirm the higher absorption rate due to organic ligand decoration on ZnO nanoparticles. The greatest solar power-to-electricity conversion efficiency (*η*) of 3.48% is achieved for the ZnO-DOL sample. It is enhanced by 3.13% as compared to that of the ZnO-based solar cell. The ZnO-DOL device exhibits a higher external quantum efficiency (EQE), responsivity (*R*_λ_), and photocurrent-to-dark current ratio; this confirms the improvement in the solar cell performance.

## Abstract 2

Efficiency improvement of the industrial scale solar cells to capture sunlight as an important renewable energy source is attracting significant attention to prevent the consumption of a finite supply of unsustainable fossil fuels.

## Graphical abstract

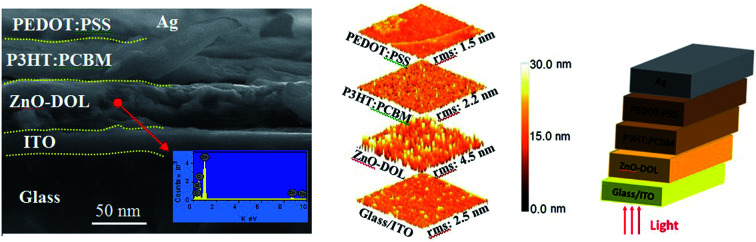

## 1. Introduction

For generating economic and environmentally friendly renewable energy, organic solar cells are a promising choice because of their appropriate size and low-temperature coating and printing process.^1^ Increasing the efficiency conversion of the conventional bulk-heterojunction solar cell architecture is possible by inserting a polymer between a transparent anode and a metal cathode.^2–4^ Exposure of these devices to air for a long time results in electrode oxidation, degradation of the active layer, and moisture diffusion through the grain boundaries and pinholes of the metal electrode.^5^ Moreover, metal deposition causes metal diffusion within the active layer, and the diffused metal probably reacts with the polymer; therefore, the semiconducting properties will be changed.^6,7^ Semiconductor-based inverted solar cells can be an approach to increase device stability. They have a similar device composition, but the electrodes are placed with a reversed polarity. In this inverted structure, the intrinsic semiconductor film is deposited on the bottom of an indium tin oxide (ITO)/glass electrode, acting like a cathode buffer layer between the bulk-heterojunction layer and the cathode to collect the electrons and block the holes. Some hole-transporting or electron-blocking materials, such as PEDOT:PSS and P3HT:PCBM, can also be added between bulk-heterojunction active layers for the purpose of facilitating hole-collection. Furthermore, a suitable high work-function metal anode is used to facilitate hole-collection. This inverted geometry also prevents PEDOT:PSS and ITO from coming in contact with each other; this causes the degradation of the function of the device because of interface chemical instabilities;^11,12^ due to this type of design, the power conversion efficiencies have been reported to be between 3 and 4 percent.^8–10^

ZnO has an application in antireflection and transparent electrode layers of solar cells because of its wide band gap (3.3 eV) nature, high exciton binding energy, and stable wurtzite structure.^13^ Because of their high surface-to-volume ratio, great sensitivity, and excellent stability, ZnO nanoparticles have attracted wide attention.

Some promising properties such as reliability, low synthesis cost, recyclability, and easy processing can be found in organic materials.^14,15^ Solar cells as an organic/inorganic dye sensitizer have the combined valuable characteristics of a conjugated or conducting polymer and the inorganic semiconductor nanoparticles with a wide band gap. The absorption band of the hybrid materials has a high-photon (sunlight) harvesting capacity. The conducting polymers can be blended with a range of inorganic semiconductors such as ZnO, TiO_2_, and CdSe. As a result, at the interface of the organic–inorganic materials, the induced charge carriers can be generated.

Therefore, for the purpose of improving the light harvesting capability and increasing the overall photon energy-to-electric conversion efficiency, imine-linked receptor-decorated ZnO-based dye-sensitized converted solar cells have been produced in the current study. The main reason for decorating the ZnO nanoparticles with the imine-linked receptors as a capping agent is to passivate the surface of ZnO nanoparticles and prevent aggregation that occurs in the case of pure ZnO. Additionally, to compare the performance of the imine-linked-decorated ZnO nanoparticles with that of pure ZnO as a cathode buffer layer, various characterization techniques have been employed.

## 2. Experimental

Herein, two kinds of solar cell samples have been prepared. Both the ZnO NPs decorated with an organic ligand (ZnO-DOL) and the other without the organic ligand act as the cathode buffer layers. The following processes have been conducted to fabricate solar cells. A detergent, deionized water, acetone, and isopropyl alcohol were used to clean the ITO-coated glass substrates (15 Ω sq^−1^) following a 5 min oxygen plasma treatment. The PEDOT:PSS was deposited onto the cathode buffer layer (ZnO and ZnO-DOL) using a spin coating technique and then annealed at 120 °C for 10 min. A chlorobenzene solution of P3HT (25 mg ml^−1^) and PCBM (15 mg ml^−1^) was then spin-coated on the PEDOT:PSS layer in a glove box followed by annealing at 160 °C for 10 min. For the completion of the inverted device structure, a Ag electrode was deposited on top of it. The abovementioned materials were purchased from Sigma-Aldrich. The process of synthesizing ZnO and ZnO-DOL nanoparticles as thin cathode buffer layers has been described hereinafter.

For synthesizing ZnO NPs by a co-precipitation method, zinc nitrate (Merck) (Zn(NO_3_)_2_·6H_2_O) and potassium carbonate (K_2_CO_3_, Merck) were used as a precursor and a precipitator, respectively. Herein, 0.03 M Zn(NO_3_)_2_·6H_2_O was dissolved in 100 ml water and added to 50 ml of a 0.05 M K_2_CO_3_ solution under magnetic stirring. The pH of the solution was adjusted to 5.5. The mixed solution was stirred at 80 °C and evaporated for 4 hours. The product was dried at 220 °C for 1 hour and then powdered into fine particles. To reach the required temperature (500 °C), the temperature of the dried precursor powder was gradually increased at a heating rate of 1 °C min^−1^, and then, the maximum temperature was maintained for 4 hours to obtain the ZnO nanoparticles.

**Fig. 1:**
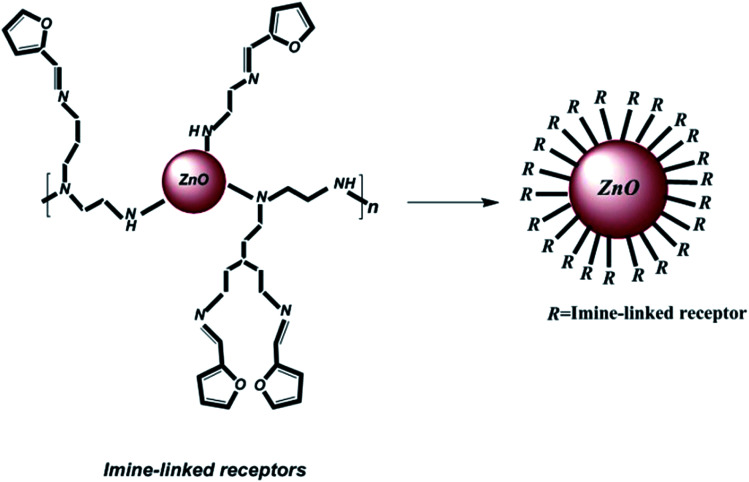
Decoration of imine-linked receptors on ZnO nanoparticles.

To prepare the organic ligand, imine-linked receptors were synthesized *via* a reaction between 2-furancarboxaldehyde (2-FC, 1, 1 mmol) and polyethyleneimine (PEI, 2, 2.5 mmol) in dry methanol. The solution was continuously refluxed for up to 6 h; after completion of the reaction, the solvent was evaporated under reduced pressure, the products were purified, and a pale yellow product was obtained. Then, the product was rinsed with methanol. Finally, for the purpose of decorating imine-linked receptors on ZnO nanoparticles as abovementioned, 804 mg (3 mmol) of the polymer compound was coupled with 100 mg ZnO nanoparticles in dry CHCl_3_, and the solution was refluxed for 15 h. Then, the obtained product was rinsed with chloroform and dehydrated under vacuum. Fig. 1 shows the schematic of the decoration of the imine-linked receptors on ZnO nanoparticles.

The thin layer of ZnO and ZnO-DOL (50 nm) as the cathode buffer layer was then spin-coated onto the ITO-coated glass and annealed in air for 5 min at 400 °C to remove the impurities and lattice disorder as well as increase the degree of crystallization.

X-ray diffraction (XRD) (D8 Advance diffractometer, Bruker, USA) was used for structural analysis. Cu Kα radiation (0.154 nm) at 40 kV and 100 mA with a step size of 0.02° and a resolution of 0.011° was employed to scan the samples from 2*θ* = 20° to 80°. To observe the deposited layers, the formation of ZnO nanoparticles, and the elemental analysis, a field-emission scanning electron microscope (FESEM, JEOLJSM 6380LA) attached with an energy dispersive X-ray spectrometer (EDX) and a high-resolution transmission electron microscope (HR-TEM, JEOL ARM200F) were used. A luminescence spectrometer (LS 55, Perkin Elmer, USA) with a 239 nm excitation wavelength was employed to obtain the room-temperature photoluminescence (PL) spectra. An FTIR spectrometer (Thermo Fisher Scientific Inc., USA) was used to distinguish the functional groups of the samples. An analysis of the surface morphology of different layers was conducted using an AFM fabricated by Seiko Instrument Inc. (SPI3800). UV-vis absorption measurement was carried out using a Shimadzu UV-3101PC double-beam spectrophotometer at room temperature. The photocurrent–voltage characteristics of the solar cells were measured using a solar simulator having a light source of AM1.5G (CEP-2000SRR, Bunkoukeiki Inc) and an incident light intensity of 100 mW cm^−2^ calibrated by a standard silicon solar cell. These conditions are experienced when the sun is at an angle of about 48° and are considered to be the best representation of the sun's spectrum on the Earth surface. The photovoltaic performance has been noted by a mask and describes the active area of the device, which is 0.20 cm^2^ and 0.12 cm^2^ in this study. Before conducting the measurement for dark *J*–*V* curves, the solar cells were illuminated by a simulated solar light for 1 min. Immediately, the devices were covered under a completely dark condition, and the *J*–*V* curves were obtained using a CHI660E electrochemical workstation in the air. The EQE spectra were obtained using a solar cell spectral response measurement system QE-R3011 (Enlitech Co. Ltd., Kaohsiung, Taiwan). The sample was excited from the ITO electrode for the electron mobility measurements and through the Ag electrode for the hole mobility measurements. A positive bias was applied to the Ag electrode in both the cases to operate the device in a reverse direction for preventing charge injection.

## 3. Results and discussion

**Fig. 2:**
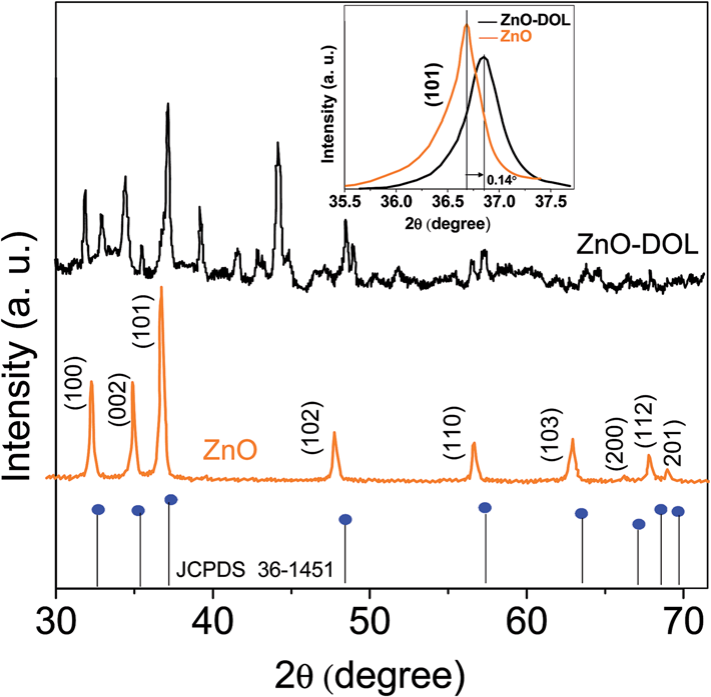
The XRD spectra of the samples with the standard (JCPDS 361451), the inset: shift in the (101) diffraction peak due to the decoration of the ZnO nanoparticles with an organic ligand.

Fig. 2 illustrates the XRD-patterns of ZnO and ZnO-DOL nanoparticles. The ZnO sample presents scattering angles (2*θ*) at 32.1°, 34.8°, 36.7°, 47.7°, 56.7°, 62.9°, and 67.7° corresponding to the reflections from the (100), (002), (101), (102), (110), (103), and (112) planes, respectively. All peaks matched with the hexagonal wurtzite structure and JCPDS card (36-1451); this indicated the purity of the ZnO nanoparticles. The imine-linked receptor-decorated ZnO nanoparticles are oriented along the planes at 2*θ* = 31.9°, 33.1°, 34.9°, 36.8°, 38.9°, 40.2°, 43.5°, 47.2°, and 56.3° angles. The difference between the spectrum of the imine-linked receptor-decorated ZnO nanoparticles and that of the non-decorated ZnO nanoparticles confirms the formation of the stable wurtzite structure of the imine-linked receptor-decorated ZnO nanoparticles. The average crystallite size of the samples is estimated using the Debye–Scherrer equation:^16^
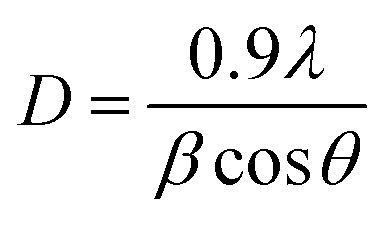
where *λ* is the X-ray wavelength (0.154 nm), *θ* is the Bragg diffraction angle, and *β* is the full width at half maximum (FWHM) of the diffraction peak. The average crystallite size of ZnO nanoparticles and ZnO-DOL is calculated to be 46 and 54 nm, respectively, which indicates the existence of a ligand *L* on the surface of ZnO nanoparticles and is in good agreement with the TEM results. The lattice parameters, the estimated crystallite size, *d*-spacing, and density of dislocation are depicted in Table 1.

**Table 1:** The XRD peak position, lattice parameters, density of dislocation, and grain size, obtained for two samples

Sample	2*θ* ± 0.01	*hkl*	*d*_*hkl*_ (nm) ± 0.0005	Lattice parameter (nm) ± 0.0005	Grain size (nm)	Density of dislocation (×10^−4^) ± 0.1
ZnO	36.70	(101)	0.2462	*a* = 0.3258	46	4.7
	34.82	(002)	0.2603	*c* = 0.5210		
ZnO-DOL	36.84	(101)	0.2458	*a* = 0.3250	54	3.4
	34.98	(002)	0.2598	*c* = 0.5204		

In small particles, surface effects are of great importance. The reduction in the lattice parameters produced by the surface tension can be considered as one of these effects. The dangling bonds on the ZnO surface interact with oxygen ions from the organic ligands, and because of the electrostatic attraction, the lattice is somewhat contracted; this is clearly confirmed by the peak shift (0.14°) to a higher angle in the XRD spectra (the inset in Fig. 2). The lattice parameters (*a* and *c*) for the hexagonal structure of both samples and density of dislocation (*δ*) are calculated using 
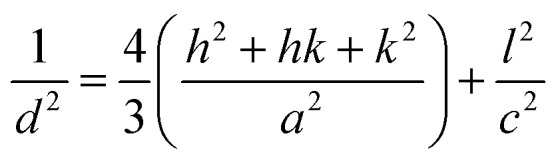
 and 
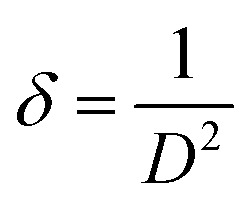
 formula, respectively, and the results are tabulated in Table 1.

**Fig. 3:**
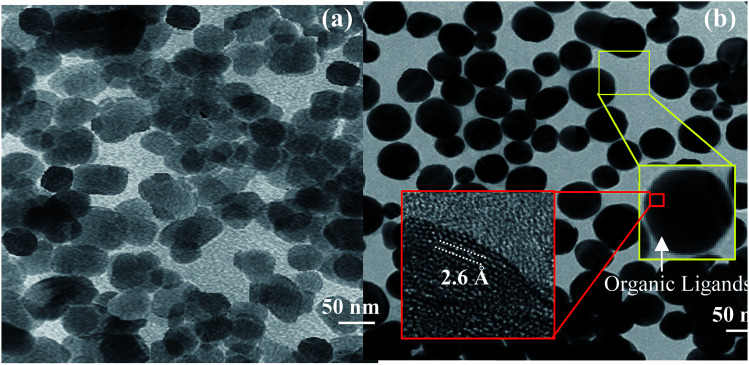
The TEM images of ZnO nanoparticles (a) and ZnO-DOL nanoparticles (b). The insets show the HR-TEM images for calculating the *d*-spacing from the areas marked with the red squares.

The TEM images of ZnO and ZnO-DOL NPs are shown in Fig. 3a and b, respectively. The existence of spherical shape nanoparticles is clearly seen. Moreover, two rectangular areas are selected to analyze the size and crystalline structure of the ZnO-DOL nanoparticles, as shown as the inset in Fig. 3b. The surface of the ZnO NPs is rough, with a few nanometers of the immune-linked receptor covering the ZnO core. The lattice fringes from ZnO NPs are visible in the HR-TEM images, and the *d*-spacing is measured to be 2.6 Å corresponding to ZnO (002), which is in correlation with the *d*-spacing extracted from XRD. By decreasing the size to the nano scale, the particles exhibit a higher relative surface area. Therefore, higher dangling bonds between adjacent particles cause agglomeration. However, the surfactants keep the particles away from each other and prevent agglomeration, as clearly seen in Fig. 3b.

**Fig. 4:**
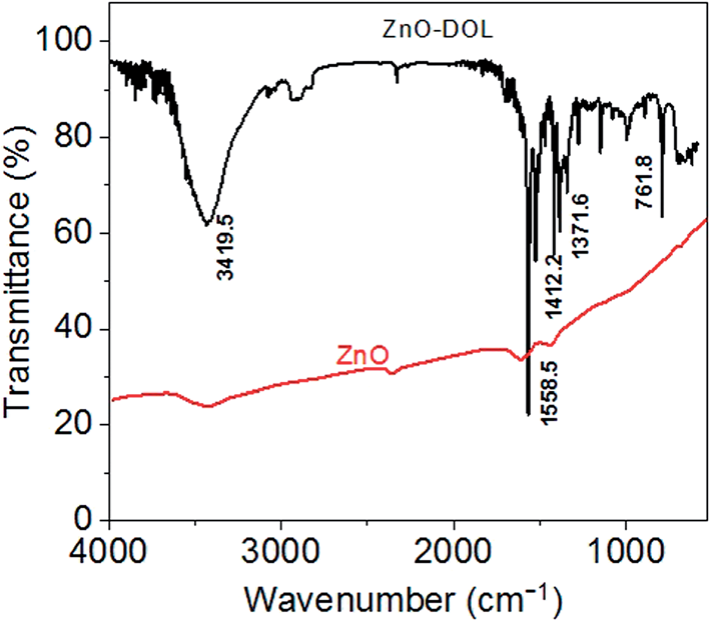
The FTIR spectra of the samples. The appearance of the peaks in the ZnO-DOL sample confirms the presence of the organic ligand on the ZnO surface.

The presence of an organic surfactant on ZnO nanoparticles is investigated using FTIR analysis. The obtained FTIR spectra for the ZnO and ZnO-DOL samples are demonstrated in Fig. 4. For uncapped ZnO, no characteristic functional groups in the low-frequency region are observed in the FTIR spectrum. However, some weak absorption peaks around 3500 and 1600 cm^−1^ can be seen, which are perhaps attributed to the hydroxyl groups originated from the hygroscopic nature of KBr used to make the pellet of the sample. On the other hand, a series of absorption peaks ranging from 500 to 4000 cm^−1^ is observed in the ZnO-DOL sample, resulting from the presence of organic groups and the chemisorbed species on the nanoparticle surface. The band around 1500 and 1600 cm^−1^ can be assigned to the vibration of the C=N group of the ligand. The appearance of the peaks at 1371 and 1412 cm^−1^ is due to the C–C stretch of the aromatic ring. Moreover, finally, the peaks around 761 cm^−1^ are associated with the C–H group of a Schiff base.

**Fig. 5:**
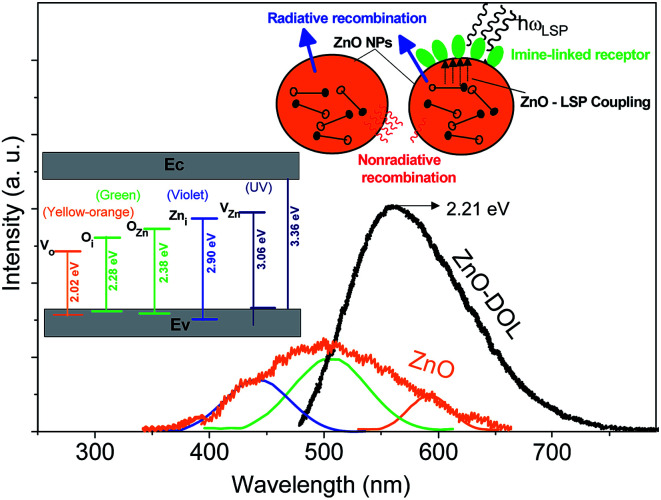
The room-temperature PL spectra of the samples. The inset demonstrates the schematic band diagram responsible for the emissions with different energies for both samples.

Herein, two types of emissions are expected to be observed from the ZnO semiconductor nanoparticles: the emission in the visible region originated from the deep level transitions and the UV emission attributed to the excitonic transitions. Deep level transitions are related to the presence of defects on the surface of ZnO nanoparticles. The existence of different electronic levels within the band gap is associated with the presence of defects, which result in electronic transitions at these levels. If the broad emissions in the visible region due to the surface defects get censored, the optoelectronic property of a metal oxide semiconductor can be improved. Fig. 5 shows the PL emission spectra of ZnO nanoparticles and ZnO-DOL nanoparticles excited at 293 nm wavelength. For the ZnO sample, the broad visible emission band is deconvoluted into three Gaussian shaped components at around 440 nm (2.81 eV), 506 nm (2.45 eV), and 594 nm (2.08 eV). The component band close to 2.81 eV is frequently labeled as a blue emission.^17^ It is attributed to the intrinsic defects (vacancies or interstitials of Zn and their complexes) in ZnO nanostructures^18^ and schematically shown in the inset in Fig. 5. The component band at about 2.45 eV is assigned to a green emission and associated with oxygen and zinc vacancies.^19^ The component bands emerged at about 2.08 eV are commonly allocated as the yellow-orange emissions in ZnO, which are linked with oxygen vacancy.^20^

The excited electron–hole pairs are recombined *via* two mechanisms: radiative and non-radiative, as shown in the inset in Fig. 5. The energy of the electron–hole pairs is conveyed in the process of recombination through a radiative mechanism. However, in the non-radiative recombination, the energy is discharged in the form of heat or other forms and is not detectable by a spectrophotometer. The energy of non-radiative recombination is bigger than the radiative energy when the pumping power density is lower than a specific threshold. This also explains the low efficiency of ZnO as a solar cell material. By contrast, when ZnO is covered with a layer of organic ligands, because of the similarity between the ZnO band gap and the surface plasmon energy of the surfactant, an extreme coupling of energy will be emerged between ZnO and surface plasmons (SP). Therefore, the energy of the electron–hole pairs is coupled into the surface plasmon of the surfactant. Moreover, the level of coupling is much greater than the level of electron–hole pair recombination.^21^ As a result, the decorated structure can efficiently couple more energy of the electron–hole pairs into the free space than the non-decorated structure (ZnO). Consequently, it boosts the intensity of photoluminescence of the ZnO-DOL sample and causes emission in a lower band gap energy (2.21 eV) as compared to the case of ZnO nanoparticles. As shown in Fig. 5, through interspaces among the organic ligands, the excited light can reach the surface of nanoparticles easily without much absorbance or scattering. Then, the energy of the electron–hole pairs is significantly coupled to the local surface plasmons of organic ligands. Finally, the local surface plasmon energy can be coupled into the free space as a radiated light because of a decrease in the wavelength vector of the local surface plasmons caused by scattering of the organic ligand.^22^

**Fig. 6:**
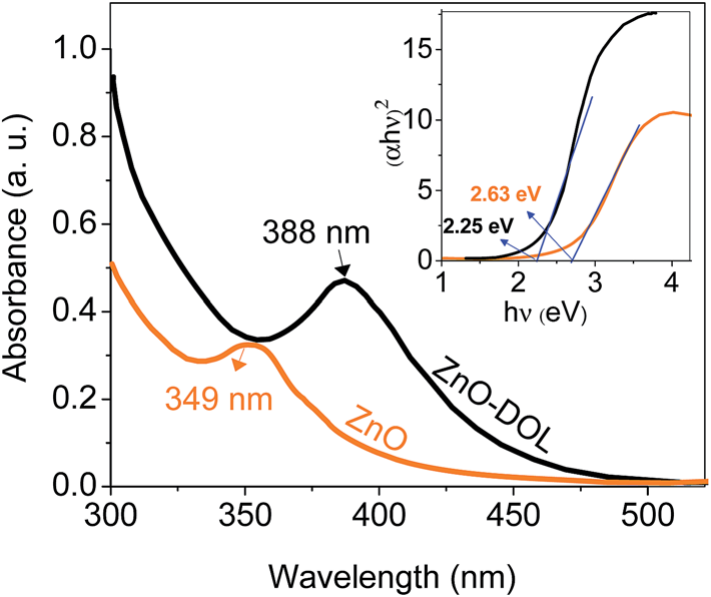
The UV-visible absorption spectra of the samples. The inset shows the Tauc's method for calculating the band gap value.

UV-vis absorption spectrometry has been carried out to determine the amount of absorbed light in two different samples, and the results are depicted in Fig. 6. The ZnO nanoparticle sample decorated with the organic ligand exhibits a higher absorption rate and a peak at 388 nm, whereas the ZnO sample exhibits a lower absorption rate with a peak at 349 nm corresponding to the exciton state of ZnO.^23^ The constant conduction and valence bands in the case of bulk materials are changed to separate the energy levels in the case of nanoparticles. Therefore, the absorption at 349 nm may be attributed to the inter-band absorption occurring between the conduction and valence bands. This red-shift from bulk to nanosize ZnO is in agreement with the PL results. In addition, the ligand that acts as a capping agent on ZnO causes another red-shift in the absorption spectra. Thus, the band gap is decreased. This decrement in the band gap is prerequisite for increasing the efficiency of the solar cell. The organic receptors are good capping agents for ZnO nanoparticles as they can efficiently passivate the surface defects and reduce the surface-related emission.^24,25^ The inset in Fig. 6 shows the Tauc plot for calculating the band gap of the sample.

**Fig. 7:**
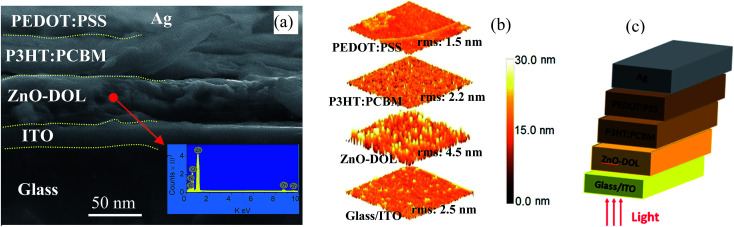
The cross-sectional FESEM image of the solar cell device with ZnO-DOL as a cathode buffer layer (a), AFM images of different layers in the flexible inverted solar cell (b), and the device configuration of the flexible inverted ZnO-based solar cell (c).

The cross-section FESEM images of the encapsulated device with a configuration of glass/ITO/ZnO-DOL/P3HT:PCBM/PEDOT:PSS/Ag are demonstrated in Fig. 7. The presence of the organic ligand on the surface of ZnO is also quantified using elemental analysis *via* point EDX spectrum (the inset in Fig. 7a). The presence of the peaks from the organic ligand (Ni and C) along with the peaks of Zn and O confirm the existence of the organic surfactant on ZnO nanoparticles. The atomic force microscopy (AFM) images of the diverse layers and the device configuration of the inverted glass solar cell are illustrated in Fig. 7b. The surface morphology indicates that the ITO/glass substrate is relatively smooth. The spin-coated ZnO-DOL nanoparticles on top indicate the slightly higher degree of surface roughness. Finally, by spin coating P3HT:PCBM and PEDOT:PSS, the surface becomes much smoother, as clearly seen in the AFM three-dimensional images.

**Fig. 8:**
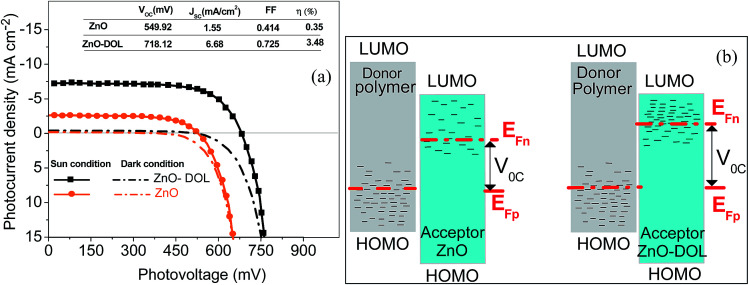
The current–voltage characteristic curve diagram of the solar cell having a ZnO and ZnO-DOL cathode buffer layer (a). The inset shows the open-circuit voltage (*V*_oc_) and the short-circuit current density (*J*_sc_), fill factor (FF), and light harvesting efficiency (*η*). Energy diagram of the polymer: ZnO and ZnO-DOL under illumination indicating the electrically active states in the band gap of both materials (b).

The typical current density *versus* voltage (*J*–*V*) curves of two complete solar cell samples based on ZnO and ZnO-DOL nanoparticles as the cathode buffer layer in the dark and under illumination are shown in Fig. 8a. In the dark, the current flow is extremely low until the contacts start to inject heavily at a forward bias for the voltages that are larger than the open-circuit voltage. The light harvesting efficiency (*η*) of two solar cells is computed using the following equation:
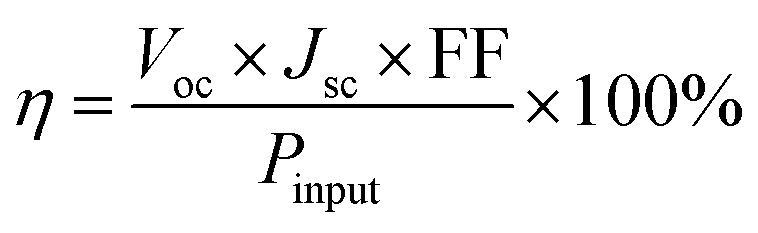
where *V*_oc_ and *J*_sc_ are the open-circuit voltage and short-circuit current density, respectively. The open-circuit voltage (*V*_oc_) is the maximum possible voltage across a solar cell. Although the factors influencing *V*_oc_ are still not fully understood, it is generally believed that the energy level offset between the HOMO of the donor and the LUMO of the acceptor minus the exciton binding energy (*E*_ex_) directly determines the value of *V*_oc_. *V*_oc_ of the multilayer heterojunction organic solar cells is given by the following formula:^26^
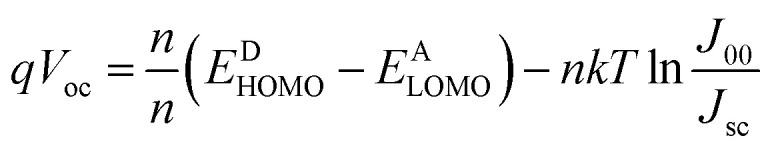


Note that the second term on the right-hand side of this equation is based on the values of the reverse saturation current density (*J*_00_). *n* is the ideal factor, and n^'^ is the ideal factor that takes into consideration the effects such as vacuum level misalignments at the heterojunction caused by energy level bending and the interface dipoles and the formation of the charge-transfer states. *k* is a Boltzmann constant, and *T* is the temperature. However, a comparison of measured *V*_oc_ with the theoretically calculated *V*_oc_ based on (*E*^D^_HOMO_ − *E*^A^_LUMO_) cannot explain the complexity of *V*_oc_. Many other factors, such as film thickness, illumination intensity, and temperature dependence, may affect *V*_oc_ of our samples, which cannot be explained by these empirical formulas. Therefore, the experimentally achieved value for our *V*_oc_ is slightly deviated from the typically reported value of 0.6 mV.^27^

The defect states and crystallinity can be deliberated as some of the additional sub-parameters that affect the energetic levels. The key factor of *V*_oc_ is the magnitude of the energy distribution of the intermediate electronic states beneath the LUMO level of the acceptor. The deposition of the high crystalline ZnO-DOL thin layer with a lower defect density on the polymer as a cathode buffer layer causes lowering of the Fermi level by increasing the available number of electronic states, as shown in Fig. 8b, and results in an increase in the *V*_oc_. In addition, the work function difference of the cathode/anode and the weight ratio of the donor and the acceptor^28,29^ are other two factors that can change the *V*_oc_. The lower work functions of the ZnO-DOL thin layer compared with those of ZnO could increase the work function difference of the cathode/anode together with crystallinity leading to an increase in *V*_oc_ from 549.92 mV to 718.12 mV.

The short-circuit current (*J*_sc_) is the current through the solar cell when the voltage across the solar cell is zero. The largest power output (*P*_max_) is determined by the point where the product of voltage and current is maximized. The filling factor is the ratio of the actual power limit to the theoretical power limit of a solar cell, which can be calculated from the division of the *P*_max_ by the product of *J*_sc_ and *V*_oc_ as follow:
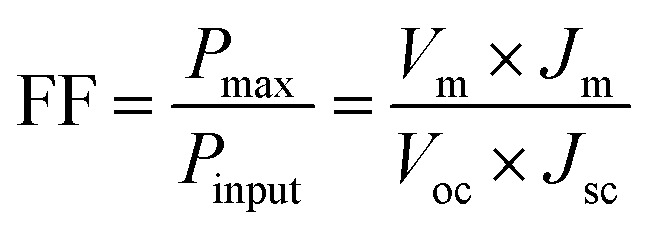
where *V*_m_ and *J*_m_ are the voltage and current density at the maximum power, respectively, corresponding to maximum output power. The photovoltaic parameters of the solar cell samples are tabulated in a table shown in the inset in Fig. 8a. It can be observed that decoration of particles with organic ligands improves the light harvesting ability of the ZnO-based solar cell. As abovementioned, the improvement in the efficiency is directly related to the higher rate of absorption that occurs in the ZnO-DOL sample.

**Fig. 9:**
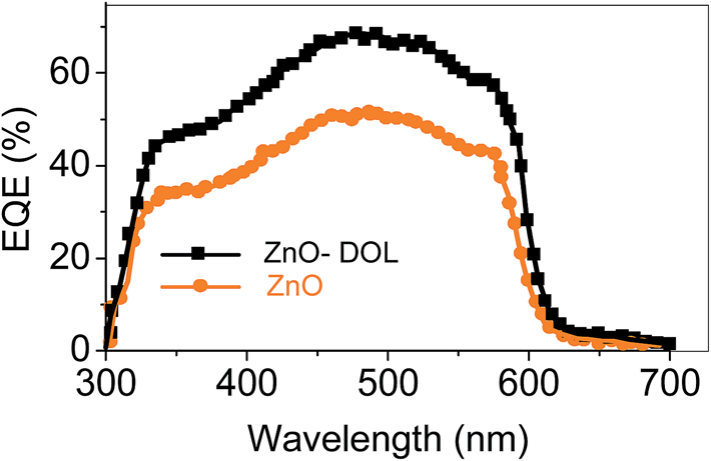
The EQE curve of the samples.

Fig. 9 shows the EQE spectra of the solar cells having ZnO and ZnO-DOL as the cathode buffer layers. In the calculation, we speculate that one absorbed photon produces one exciton in the active layer and one exciton divides into two free charges including an electron and a hole. Then, one electron or hole is collected by a cathode or an anode, respectively. As a result, the number of photons absorbed in the active layer can be used as a substitute for the maximum possible short-circuit current density, and the EQE can be simplified as the ratio of the number of photons absorbed in the active layer to the number of the incident photons. EQE can be calculated by the following equation:
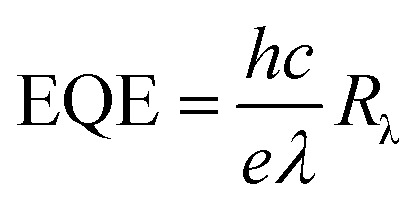
where *λ* is the excitation wavelength, *h* is the Planck's constant, *c* is the velocity of the light, *R*_λ_ is the responsivity, and *e* is the electronic charge.^30^ We can observe that the EQE of the ZnO-DOL device is generally higher than that of ZnO over all the absorption range; this suggests an enhanced light harvesting and causes higher efficiency by extracting the electrons from the organic-semiconductor interface. The EQE of both samples is decreased in the ultraviolet (UV) region because the ZnO layer absorbs UV light and plays the role of a light filter, which is in agreement with the absorption spectra shown in Fig. 6.

The responsivity (*R*_λ_) is defined as the photocurrent per unit of the incident optical power and calculated using the following equation:^30^
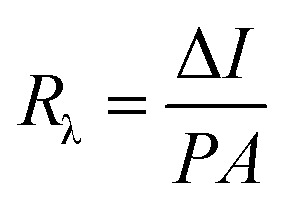
where Δ*I* is the difference between the photo-excited current and dark current, *P* is the power density irradiated on the sample, and *A* is the irradiated area of the device. As shown in Table 2, ZnO decorated with the organic ligand exhibits a higher amount of *R*_λ_ and EQE at 492 nm light wavelength. The *R*_λ_ and EQE of the ZnO-DOL-based device are 30.4 A W^−1^ and 70% at 492 nm, respectively, which are considerably higher than those of the ZnO-based device (∼10.2 A W^−1^ and ∼50%, respectively). The greatly enhanced photocurrent and photocurrent-to-dark current ratio suggests that ZnO-DOL has great advantages in improving the performance of the solar cell.

**Table 2:** Comparison of the dark current, photocurrent, *R*_λ_, and EQE for ZnO and ZnO-DOL devices

Sample	Light of detection (nm)	Dark current: *I*_D_ (nA)	Photo current: *I*_I_ (mA)	*I*_I_/*I*_D_	*R*_λ_ (A W^−1^)	EQE (%)
ZnO	492	0.52	2.39	4.59	10.2	50
ZnO-DOL	492	1.08	6.52	6.03	27.4	70

**Fig. 10:**
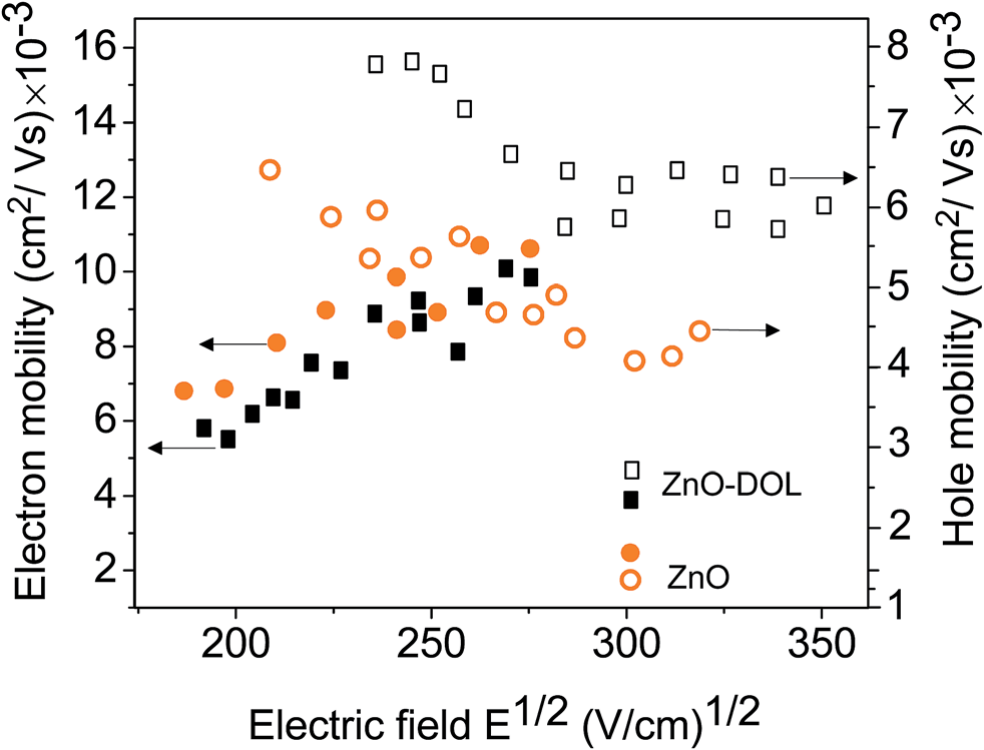
The electron and hole mobility of two different samples, represented by different symbols.

Fig. 10 illustrates the electron and hole mobility of two devices as a function of the applied electric field. The time of flight measurement technique is a widely used method for determining the mobility. It measures the mobility perpendicular to the substrate at a low charge density. Obviously, the electron mobility of the ZnO device is higher than that of the device with ZnO-DOL. This is due to the highly uniform and polycrystalline nature of the thin ZnO sample.^31^ In the thicker ZnO-DOL sample, the transport length of the charge carriers can be increased, and as a result, the recombination possibilities are accordingly enhanced, and thus, the electron mobility is reduced. However, the hole mobility of the ZnO-DOL sample is higher than that of the ZnO sample due to the following reasons.

Basically, the mobility of holes in these organic semiconductors is related to the level spectrum of the transport states. The p-type doping requires the transfer of an electron from the filled HOMO of the host to the LUMO of the dopant at either no or only a little bit expense of energy. While the n-type doping needs more energy because the HOMO of the dopant has to be close to the LUMO of the host to promote this type of doping. This puts serious constraints on the mutual energy levels and makes the mobility of the holes much more low-cost in energy than that for the electrons (or better polarons due to the importance of the phonons in these materials). The power efficiency of the solar cell is limited by the recombination of the carriers. These two opposite behaviors of the electron and hole mobility in two different samples balance with each other; this results in the higher power conversion efficiency of the ZnO-DOL sample.

## 4. Conclusion

Dye-sensitized solar cells having wurtzite ZnO and ZnO-DOL nanoparticles as the cathode buffer layers are prepared by co-precipitation and spin coating methods. In this study, the effects of imine-linked decoration of ZnO nanoparticles on the performance of an organic ligand-based solar cell have been investigated. The size of the nanoparticles is measured using the Scherrer equation and found to be ∼46 and ∼54 nm for ZnO and ZnO-DOL, respectively. The red-shifts in the band gap energy from both bulk to ZnO nanoparticles and ZnO nanoparticles to ZnO-DOL nanoparticles are attributed to the deep level transition, the plasmon energy of the surfactant, and the coupling of ZnO with the local surface plasmons energy. The higher emission intensity and lower band gap for ZnO-DOL confirm the better solar cell performance due to higher generation of photocarriers with a lower photon energy, which are prerequisite for a solar cell. The higher rates of absorption, electricity conversion efficiency, EQE, and *R*_λ_ for the ZnO-DOL sample indicate its higher performance as compared to that of the ZnO device. Our systematic, low-temperature processed, inverted solar cells with a high work function metal and a high efficiency are important to develop low-cost, large-scale, and roll-to-roll printable solar cells.

## Conflicts of interest

There are no conflicts to declare.

## Supplementary Material
